# MicroRNAs and Cardiovascular Diseases

**DOI:** 10.1155/2015/682857

**Published:** 2015-02-01

**Authors:** Tsuyoshi Nishiguchi, Toshio Imanishi, Takashi Akasaka

**Affiliations:** ^1^Department of Cardiovascular Medicine, Wakayama Medical University, 811-1 Kimiidera, Wakayama 641-8509, Japan; ^2^Department of Cardiovascular Medicine, Hidaka General Hospital, 116-2 Sono, Gobo, Wakayama 644-8655, Japan

## Abstract

Coronary artery diseases (CAD) and heart failure have high mortality rate in the world, although much progress has been made in this field in last two decades. There is still a clinical need for a novel diagnostic approach and a therapeutic strategy to decrease the incidence of CAD. MicroRNAs (miRNAs) are highly conserved noncoding small RNA molecules that regulate a large fraction of the genome by binding to complementary messenger RNA sequences, resulting in posttranscriptional gene silencing. Recent studies have shown that specific miRNAs are involved in whole stage of atherosclerosis, from endothelium dysfunction to plaque rupture. These findings suggest that miRNAs are potential biomarkers in early diagnosis and therapeutic targets in CAD. In the present review, we highlight the role of miRNAs in every stage of atherosclerosis, and discuss the prospects of miRNAs in the near future.

## 1. Introduction

Cardiovascular diseases (CVD), especially coronary artery diseases (CAD) are the leading cause of death in the developed world. Atherosclerosis is the basis of CAD, and there are many known conditions promoting atherosclerosis such as diabetes mellitus, hypertension, dyslipidemia, and smoking. Patients suffering from CAD usually have these coronary risk factors for long time. In other words, CAD is the terminal phase of atherosclerosis. Enormous amount of effort has been devoted to controlling these conditions to decrease the incidence of CAD in past two decades.

Much progress has been made in the clinical settings; antiplatelet drugs, statins, angiotensin converting enzyme inhibitors, angiotensin receptor blockades, and antidiabetes mellitus drugs decrease the incidence of CAD. In addition, primary percutaneous coronary intervention dramatically improves the prognosis in patients with acute myocardial infarction (AMI). Nevertheless, CAD, especially AMI and heart failure due to ischemic cardiomyopathy are still critical problems in the world and so is exploring novel approaches which can advance the current therapeutics strategies.

In recent years, a large number of data suggest that noncoding RNAs (ncRNAs), which are not responsible for protein coding, play an important role in development of various disorders, including cardiovascular disease. Among ncRNAs, microRNAs (miRNAs, miR) are the best-characterized molecules which are small, approximately 22-nucleotide sequence of RNA.

MiRNAs are encoded in the genomes of nucleated cells and are transcribed into primary miRNAs (pri-miRNAs) that are transformed into miRNAs precursors (pre-miRNAs) by Drosha, a nuclear RNase III. After being processed by Drosha, pre-miRNAs are exported to cytosol and processed by the RNase III Dicer that, in the end, generates miRNAs. Mature miRNAs are involved in ribonucleoprotein (RNP) complexes containing Argonate 2 (Ago 2), a crucial component of RNA-induced silencing complex (RISC) [1–3].

MiRNAs regulate gene expression of messenger RNA (mRNA) at the posttranscriptional level through transcript degradation or translational repression by binding to the 3′ UTR of the target mRNA. To date, more than 2,500 miRNAs have been reported in humans (miRBase20 http://www.miRbase.org/). On the other hand, each miRNA has dozens to hundreds of target mRNAs for its permissive binding requirement to mRNA, suggesting that an mRNA is regulated by multiple miRNAs.

MiRNAs are involved in every stage of biological process of atherosclerosis, that is, endothelium dysfunction, cellular adhesion, plaque development, and plaque rupture. MiRNAs in CAD is rapidly emerging field, and new findings are continuously emerging, it is hard to update the latest knowledge.

Thus, this review summarizes the recent findings of miRNAs every stage of CAD and discusses the prospects of miRNAs in the near future.

## 2. Time Course of Coronary Artery Disease and Associated miRNAs in Every Stage

### 2.1. Endothelial Dysfunction and miRNAs

The first step in the development of atherosclerotic lesion is dysfunction of endothelium. Endothelium consists of a single layer of endothelial cells (ECs) and maintain vascular homeostasis. ECs control a balanced release of various vasoconstriction or vasodilation chemical mediators such as endothelin-1 (ET-1) or endothelial nitric oxide synthase (eNOS). ECs also regulate the coagulation and fibrinolysis systems or various pro- or anti-inflammatory mediators [4].

Dysfunction of ECs occurs when factors that favor a vasoconstriction, procoagulation, and proinflammation become predominant. There are many conditions causing dysfunction of ECs, such as diabetes mellitus, dyslipidemia, hypertension, smoking, and aging. As a result of dysfunction of ECs, oxidative stress and proinflammatory mediators become predominant in these individuals and cause following atherosclerosis. MicroRNAs regulating endothelial functions are summarized in Table 1.

#### 2.1.1. miRNAs in ECs Associated with Diabetes Mellitus

To maintain appropriate blood glucose level is crucial for cell homeostasis and survival. Glucose metabolism is strictly regulated. There are several peptides, including insulin and glucagon, which regulate glucose metabolism.

Type 2 diabetes mellitus (T2D) is the most common metabolic disorder worldwide [5]. T2D is characterized by chronic hyperglycemic state with low insulin production by pancreatic *β* cells and/or low responsiveness by insulin target tissues, mainly liver, adipose tissue, and skeletal muscle. It is also known that insulin itself has endothelium protective functions. Insulin promotes endothelium-dependent relaxation through an increase of eNOS production via activation of phosphatidylinositol-3 kinase (PI3K) and Akt kinase pathways [6, 7]. eNOS facilitates the production of nitric oxide (NO) that is important in preventing atheroma formation. In addition, insulin also activates mitogen activated protein kinase (MAPK) pathway. As well as PI3K pathway, MAPK pathway plays central role in insulin-signaling and promotes the expression of ET-1 in ECs [8, 9]. In individuals under diabetes state, insulin resistance impairs the activity of PI3K pathways and downregulates the production of NO in ECs. Interestingly, MAPK pathway is not affected under pathological state in insulin resistance [10]. Such selective resistance to insulin results in decrease production of NO and increase production of ET-1 and leads to dysfunction of ECs. Thus, it is now well accepted that endothelial dysfunction and insulin resistance are frequently comorbid states and responsible for the increased cardiovascular risks in patients with T2D [8].

*MiR-126*. MiR-126 is one of the most studied miRNAs in vascular biology. Zampetaki et al. have reported that circulating level of miR-126 is reduced in patients with T2D [11]. MiR-126 expressed in human hepatocytes is involved in the development of insulin resistance through the inhibition of insulin receptor substrate 1 (IRS1) [12]. Furthermore, miR-126 negatively regulates the vascular endothelial growth factor (VEGF) pathway, including the sprouty-related protein (SPRED1) and phosphoinositol-3 kinase regulatory subunit 2 (PIK3R2/p85-*β*) and plays an important role in maintaining vascular integrity, angiogenesis, and wound repair [13, 14]. Recent study shows that miR-126 is predominantly expressed in endothelial microparticles (EMPs), and EMPs play crucial role in EC migration and repair via SPRED1 pathway. Interestingly, the amount of miR-126 in EMPs is significantly lower in diabetic conditions, and the capacity of endothelial repair is reduced* in vivo* and* in vitro* [15].

MiR-126 is known to be divided into two subtypes, miR-126-3p and miR126-5p [13, 16]. Recently, miR-126-5p is found to maintain a proliferative reserve in ECs through suppression of the Notch1 inhibitor delta-like 1 homolog (Dlk1) and prevents atherosclerotic lesion formation in ApoE^−/−^ mice [17].

*MiR-320*. The function of miR-320 is controversial. Wang et al. reported that high glucose induced miR-320 expression in microvascular ECs in diabetic rats [18]. Moreover, the proliferation and migration of ECs were improved after transfection of the miR-320 inhibitor, indicating that miR-320 is negatively regulating the ECs proliferation [18].

On the other hand, circulating miR-320 is found to be decreased in diabetic patients [11]. In addition, Feng and Chakrabarti recently reported that high glucose exposure decreased the expression of miR-320 but increased the expressions of ET-1, VEGF, and fibronectin (FN) in human umbilical vein endothelial cells (HUVECs) [19]. Transfection of miR-320 mimics decrease of the expressions of ET-1, VEGF, and FN in HUVECs treated with high concentrations of glucose [19].

*MiR-1*. MiR-1, specifically expressed in cardiac and skeletal muscle, has widespread biological effects in the development of cardiovascular systems [20–24]. Overexpression of miR-1 during embryonic period resulted in developmental arrest due to thin-walled ventricles and heart failure in mice [21]. On the other hand, miR-1 knockout mice resulted in ventricular septal defect in the half, and the remaining majority without structural abnormality resulted in sudden death due to abnormal cardiac conduction or repolarization [20].

MiR-1 plays a crucial role in the development of cardiovascular diseases as well as in cardiogenesis. Yang et al. revealed that the expression of miR-1 was upregulated in patients with CAD and rats with experimental myocardial infarction [22]. Furthermore they found that miR-1 regulates cardiac arrhythmogenic potential by targeting several ion channel genes [22]. In addition, miR-1 is associated with cardiac hypertrophy [23, 25, 26]. Sayed et al. showed that the expression of miR-1 was immediately downregulated in cardiac hypertrophy model mice with aortic constriction [23]. They also demonstrated that miR-1 regulates cardiac hypertrophy by negatively regulating the necessary genes for cardiac hypertrophy such as Ras GTPase-activating protein and cyclin-dependent kinase 9 [23].

MiR-1 also regulates the expression level of ET-1 through targeting ET-1 gene (EDN-1) [27]. Recent study shows that miR-1, similarly to miR-320, regulates glucose induced upregulation of ET-1 in human retinal ECs and HUVECs [28]. High glucose exposure decreased the expression of miR-1 but increased the expression of ET-1 in ECs, and these patterns were inhibited by transfection of miR-1 mimics [28]. Decreased miR-1 level and increased ET-1 level were also demonstrated in animal diabetic models [28]. Dysfunctions of ECs in diabetes may be related to the downregulation of miR-1/ET-1 pathway in part.

*MiR-503*. Recent studies show that miR-503 in ECs is upregulated in diabetic animals and human [18, 29]. MiR-503 plays an important role in cell cycle quiescence and differentiation in G1-to-S translation through the degradation of cdc25A and cyclin E [29–31]. The expression of miR-503 is remarkably high, while the expression of cdc25 protein is inversely correlated in diabetic conditions. Hence, migration, proliferation, and tube formation of ECs are inhibited, resulting in dysfunction of ECs.

In fact, expression of miR-503 in ECs is upregulated in diabetic patients with critical limb ischemia, and circulating miR-503 level is significantly increased in these patients in comparison with controls. These results indicate that miR-503 can serve as a potential biomarker of ongoing ischemia [29].

#### 2.1.2. miRNAs in ECs Associated with Dyslipidemia

Dyslipidemia is well known to be a major cause of CAD. Now, lowering low-density lipoproteins (LDL) therapy using statins is a completely established strategy to reduce the risk of CAD. Former studies revealed that high-density lipoproteins (HDL) or LDL cholesterol affect endothelial cells and regulate the expression of cellular adhesion molecules such as intracellular adhesion molecule (ICAM) and monocyte chemoattractant protein-1 (MCP-1) [32]. However, there are few studies investigating the direct relationship between miRNAs in ECs and dyslipidemia.

*MiR-223*. Vickers et al. found that human HDL contains miRNAs and transported them to other cells, indicating the new mechanism of intracellular signaling [33]. They found that miR-223 was the most abundant in HDL. Interestingly, HDL-miRNA profiles in patients with familial hypercholesterolemia were significantly different from those of controls, suggesting the importance of quality as well as quantity of HDL. In addition, they showed that HDL-miRNA from atherosclerotic subjects induced differential gene expression in hepatocytes [33]. Furthermore, same group reported that miR-223 in HDL was transported to ECs and decreased expression of ICAM-1 in ECs, indicating the anti-inflammatory function of HDL regulated by miRNA [34].

#### 2.1.3. miRNAs in ECs Associated with Hypertension

Hypertension impairs endothelial functions and is an established risk factor for CAD; however, little is known about the involvement of miRNAs in the pathogenesis of hypertension. Several studies showed that specific miRNAs are differentially expressed in patients with hypertension compared to controls in plasma or kidney [35, 36].

*MiR-155*. Activation of the renin-angiotensin-aldosterone system (RAAS) is a major cause of hypertension. Each step in this system is the target to lower blood pressure in patients with hypertension, and many drugs are now available. Angiotensin II (Ang II) is the major bioactive product in the system and regulates multiple aspects of EC function [37, 38]. Ang II induces the expression of adhesion molecules, such as VCAM, ICAM, MCP-1, and Fms-like tyrosine kinase 1 (FLT-1) through the activation of transcription factor Ets-1 in ECs [38–40]. Recent study showed that Ets-1, induced by Ang II, is negatively regulated by both miR-155 and miR-221/222 which are highly expressed in ECs. In addition, overexpression of miR-155 or miR-221/222 in HUVECs downregulates Ang II-induced expressions of VCAM-1, MCP-1, and FLT-1 mRNA [41]. In addition, miR-155 is found to regulate the expression of angiotensin II type 1 receptor negatively in HUVECs and reduce Ang II-induced ERK1/2 phosphorylation and activation [42, 43]. Moreover, miR-155 mediates endothelial inflammation and decreases nuclear factor-kappa B (NF-*κ*B) p65 and adhesion molecule expression such as ICAM-1 or VCAM-1 in ECs treated by TNF-*α* [44]. These findings indicate that miR-155 have protective functions in ECs. Consistently, circulating miR-155 is reduced in patients with CAD compared to healthy controls [45].

On the other hand, several studies reported the adverse functions of miR-155 on ECs and atherosclerosis [46, 47]. Decrease of NO or eNOS in ECs is thought to be a hallmark of endothelium dysfunction. Sun et al. reported that miR-155 regulates the expression of eNOS. They found that overexpression of miR-155 decreased, whereas inhibition of miR-155 increased, eNOS expression and NO production in HUVECs and acetylcholine-induced endothelium-dependent vasorelaxation in human internal mammary arteries [46].

These apparent contradiction results may be due to the nature of miRNAs; that is, an miRNA targets several mRNAs and different pathways. Further investigations on whether miR-155 finally has EC protective or invasive function are needed.

#### 2.1.4. miRNAs in ECs Associated with Smoking

There are many studies investigating the association of smoking and miRNAs; however, most of them are concentrating in respiratory field [48]. There are few data investigating the relationship between smoking and miRNA in CAD to date. MiR-223 (detailed function of miR-223 is described in Section 5) is one of the predicted miRNAs associated with smoking and CAD [49]. The expression of miR-223 is significantly downregulated in individuals with smoking habitant compared to those without one even in young healthy populations [49].

Although there are not any studies investigating the direct relationship between smoking and miRNAs on endothelium functions, it is obvious that smoking increases superoxide or reactive oxygen species (ROS) and impairs endothelial functions [50, 51]. It seems to be promising that smoking is associated with several miRNAs and involved in the development of CAD, further investigations are needed.

#### 2.1.5. miRNAs in ECs Associated with Aging

Aging is shown to be an independent risk factor in atherosclerotic diseases including CAD in epidemiological study [52]. Senescent cells increase during aging, and accumulation of senescent cells may contribute to age-related disease [53, 54]. Senescence of ECs is considered an important factor of CAD [55]. Expression of senescence-associated *β*-galactosidase (SA*β*-gal) was increased in ECs in atherosclerotic coronary artery, and senescent human aortic ECs (HAECs) exhibited increased ICAM-1 expression and decreased eNOS activity [55]. Silent information regulator 1 (SIRT-1) is also a considered molecule that exerts protective effects against endothelial senescence through increasing eNOs activation [56, 57].

*MiR-217*. MiR-217, expressed in ECs, is considered to be associated with cell senescence. Menghini et al. reported that overexpression of miR-217 induces senescence whereas inhibition of miR-217 delays senescence in several human ECs [58]. They found that miR-217 induces a premature senescent-like phenotype, which is higher expression of SA*β*-gal positive cells and leads to an impairment in angiogenesis through the inhibition of SIRT-1. They also found that miR-217 which was highly expressed in human atherosclerotic plaques negatively correlate with reduced level of SIRT-1 [58].

*MiR-34a*. MiR-34a is well studied in the field of cell apoptosis and oncogenesis [59–61]. MiR-34a induces the expression of p53, well known as a tumor suppressor gene, through inhibition of SIRT-1 pathways [59]. Interestingly, p53 regulates the expression of miR-34a, indicating the positive feedback loop between p53 and miR-34a [60].

Ito et al. found that miR-34a, similarly to miR-217, increases in senescent HUVECs and overexpression of miR-34a induces cell senescence through modulation of SIRT-1 [62]. MiR-34a also affects endothelium progenitor cells (EPCs), and induces cell senescence [63]. Tabuchi et al. showed that expression levels of miR-34a were higher in patients with CAD than those without CAD, whereas levels of SIRT-1 were lower in patients with CAD [64]. Interestingly, they treated the patients with two statins (atorvastatin or rosuvastatin) and found that only patients treated with atorvastatin had markedly decreased miR-34a levels and increased SIRT1 levels, whereas patients treated with rosuvastatin showed no change in these levels [64]. Recent study showed that shear stress also increased the expression of miR-34a in EPCs [65].

*MiR-146a*. MiR-146a may be associated with senescence of ECs. MiR-146a was found to be downregulated in HUVECs during aging [66]. Loss or gain of function experiments showed that NADPH oxidase-4 (NOX4) was the target protein of miR-146a. Moreover, miR-146a overexpression decreased SA*β*-gal positive cells, whereas inhibition of miR-146a resulted in a further increase of SA*β*-gal positive cells, suggesting that miR-146a had a causal role in cell senescence via NOX4 regulation [66].

#### 2.1.6. miRNAs in ECs Associated with Blood Flow

Mechanical forces associated with blood flow, known as shear stress, play an important role in regulating vascular signaling and gene expression in ECs. It is widely accepted that low shear stress is crucial to maintain vascular homeostasis, whereas high shear stress impairs EC functions.

*MiR-21*. MiR-21 is shown to be highly expressed miRNA in ECs. Weber et al. reported that miR-21 was induced by unidirectional shear stress in HUVECs, and ECs overexpressing miR-21 had decreased apoptosis through downregulation of the phosphatase and tensin homologue (PTEN) expression but increased eNOS phosphorylation and NO production through PI3K/Akt/eNOS pathway [67]. Overexpression of miR-21 reduced EC proliferation, migration, and tubulogenesis through repression of Rho-B, whereas miR-21 inhibition resulted in opposite effects, indicating the antiangiogenic function of miR-21 [68]. MiR-21 is known to exist in circulating plasma, and a former study reported that the expression of miR-21 in plasma was reduced in patients with T2D compared to controls [11].

Contrary to these data, Zhou et al. reported that oscillatory shear stress induced the expression of miR-21 in HUVECs, and overexpression of miR-21 enhanced the expression of VCAM-1 and MCP-1 and the consequential adhesion of monocytes to ECs through the repression of peroxisome proliferators-activated receptor-*α* (PPAR*α*) [69]. In addition, miR-21 in angiogenic progenitor cells (APCs) is shown to mediate dysfunction of APCs* in vitro* and* ex vivo* study [70]. APCs are known to be early outgrowth EPCs, and the expression pattern of miRNAs is highly similar to HUVECs or human coronary artery ECs. The expression level of miR-21 was increased in patients with CAD compared to controls [70].

*MiR-181b*. MiR-181b is a potent regulator of nuclear factor *κ*B (NF-*κ*B) signaling in ECs by targeting importin-*α*3, a protein that is required for nuclear translocation of NF-*κ*B [71]. MiR-181b was shown to decrease the importin-*α*3 expression and increase the expressions of adhesion molecules such as VCAM-1 and E-selectin in HUVECs* in vitro* and* in vivo.* [71]

*MiR-10a*. MiR-10a contributes to the regulation of proinflammatory endothelial phenotypes in atherosusceptible regions. In atherosusceptible endothelium, expression of miR-10a was decreased, whereas expression of Homeobox A1 (HOXA1), known target of miR-10a was increased in adult swine [72]. MiR-10a regulates NF-*κ*B signaling in ECs by binding in the 3′ UTR of mitogen-activated kinase kinase kinase 7 (MAP3K7; TAK1) and *β*-transducin repeat-containing gene (*β*TRC). NF-*κ*B activation and expression of VCAM-1 and MCP-1 were significantly upregulated in miR-10a knockdown human aortic endothelial cells [72].

## 3. Cellular Adhesion/Plaque Development and miRNAs

One of the pathological hallmarks of atherosclerosis is the accumulation of cholesterol by macrophages. Under the conditions of endothelial dysfunction, cytokines, and vasoactive peptides, including tumor necrosis factor *α* (TNF-*α*), and Ang II induce a variety of adhesion molecules. These molecules mediate the adhesion of leukocytes, mainly monocytes, to vascular endothelium, and migration into the vessel wall. In the vessel wall, monocytes under the stimuli such as macrophage colony-stimulating factor (M-CSF), which can increase their expression of scavenger receptors, differentiate to macrophages. Macrophages with scavenger receptors mediate the uptake of modified lipoprotein particles and development of foam cells and release proinflammatory cytokines and growth factors. Furthermore, vascular smooth muscle cells (VSMCs) migration and proliferation are also crucial for progression of atheroma into more complex plaques [73].

### 3.1. Monocyte/Macrophage and miRNA

Circulating monocytes are known to be heterogeneous and can be divided at least into two major populations according to their surface expression of CD14 and CD16, described as classical or inflammatory CD14^++^CD16^−^ monocyte and nonclassical or resident CD14^++^CD16^+^ monocyte in human. These monocyte subsets are different in the expression of chemokine and receptors reflecting distinct mechanisms of recruitment or functions in the development of atherosclerosis [74, 75]. Recent study showed that miRNA expression profiles are different in classical and nonclassical monocyte subsets [76]. MiRNAs in monocyte are carried into the vessel wall through monocytes adhesion. In the same study, expression levels of miRNAs between atherosclerotic plaque and healthy arteries in human were compared and revealed that miR-99b and miR-152 were coexpressed in plaque tissue and classical monocyte, indicating that these miRNAs within plaque may be originated from classical monocyte [76].

Similarly to monocyte subsets, macrophage has also several subsets such as M1 or M2 macrophage. M1 macrophages (classically activated macrophages) and foam cells, secreting proinflammatory molecules such as IL-1*β*, MMP-9, or TNF-*α*, play central role in plaque progression, thinning fibrous cap, and plaque destabilization, while M2 macrophages (alternatively activated macrophages) are associated with anti-inflammatory responses with IL-10 or TGF-*β* [77, 78]. It is shown that several miRNAs including miR-125a, miR-155^*^, and miR-26a^*^ affect the differentiation toward M1 macrophage [79, 80]. MiR-125a-5p and miR-155 are shown to downregulate the lipid uptake in oxidized LDL (ox-LDL) stimulated human macrophages [81, 82].

*MiR-155*. MiR-155, a typical multifunctional miRNA, is expressed not only in ECs but also in VSMCs, dendritic cells, lymphocytes, neutrophils, and macrophages [83]. Functions of miR-155 in plaque development are still controversial, same as previously described in the section of ECs dysfunction [83]. Several studies demonstrated that expression of miR-155 in atherosclerotic plaque or macrophage was induced by ox-LDL [47, 82]. Donners et al. demonstrated that deficiency of miR-155 enhanced atherosclerotic plaque development and decreased plaque stability through recruitment of the inflammatory monocytes and reduction of the resident monocytes in ApoE^−/−^ mice [84]. Zhu et al. also demonstrated that miR-155 contributes to the prevention of atherosclerotic plaque formation and progression through the inhibition of MAPK pathway in mice [85]. Thus, miR-155 is thought to be a part of negative feedback loop, which downregulates inflammatory response and development of atherosclerotic plaque.

On the other hand, Nazari-Jahantigh et al. reported that miR-155 deficiency reduced plaque size and number of lesional macrophages in atherosclerotic ApoE^−/−^ mice, and miR-155 directly promotes atherosclerosis by repressing Bcl6 in macrophages [47].

These contradictory results are from the complexity of miR-155 mediated regulation of atherosclerosis.

### 3.2. VSMCs and miRNA

VSMCs provide structural support to the vasculature and contribute to control blood pressure and blood flow through highly regulated contractile mechanisms. Although a certain number of VSMCs exist within the intima, the majority of VSMCs are distributed in media in the healthy vessel wall. Otherwise, in atherosclerotic lesion, a significant number of VSMCs are found in intima. The functions of VSMCs are different according to the location in which they exist; concisely speaking, VSMCs in media express proteins involved in the contractile functions such as smooth muscle myosin heavy chain (SM-MHC) and smooth muscle *α*-actin (SM-*α*A), whereas VSMCs in intima have a greater synthetic capacity for extracellular matrix, proteases, and cytokines [86]. The modulation of VSMCs from contractile to synthetic, in other words, from differentiation to dedifferentiation phenotype which is accompanied by accelerated VSMCs proliferation, plays a crucial role in the pathogenesis of a variety of proliferative vascular diseases including CAD. Gene expressions of VSMCs are regulated by multiple molecules or pathways, such as serum response factor (SRF) and its cofactors, RhoA, Notch signaling, and platelet derived growth factor (PDGF) pathway [87]. Among them, SRF and myocardin are the major regulators of smooth muscle gene expression [88, 89]. SRF and myocardin complex induce the expression of contractile smooth muscle proteins such as SM-MHC [89]. Recent studies have shown the specific miRNAs as significant mediators of the modulation of VSMCs phenotype by targeting these transcription factors [90].

#### 3.2.1. MicroRNAs Mediating a Phenotype Switch toward Contractile VSMCs

*MiR-143/145*. MiR-145 is expressed in a gene cluster together with miR-143. MiR-145 is highly expressed in VSMCs in the normal vascular wall and has been found to control the phenotype by upregulation of VSMCs-specific proteins such as SM-MHC, while the expression is dramatically reduced following vascular injury, during atherosclerosis* in vivo* and* vitro*, and in human aortic aneurisms [91–95]. Decreased miR-145 following vascular injury or induction of miR-145 inhibitor induces downregulation of myocardin expression, and phenotype switching toward synthetic VSMCs through upregulation of Krüppel-like factor 5 (KLF5), the target gene of miR-145. Then, VSMCs switching toward synthetic phenotype increase proliferation and result in increased neointimal lesion formation [92]. Experiments using miR-143/145 KO mouse model showed that genetic deletion of miR-145 was associated with impaired stress fiber expression, reduced vessel wall thickness, and increased endoplasmic reticulum which is typical for synthetic VSMCs [96]. Taking these observations into account, miR-143/155 cluster is thought to be a protective miRNA against atherosclerosis. Supporting this, an observational study reported that circulating miR-145 was significantly reduced in patients with CAD compared with healthy controls [45].

Of note, recent study showed the communication between ECs and VSMCs using miR-143/145 [97]. It is interesting that the transfer of miR-143/145 from ECs to VSMCs through microvesicles reduces atherosclerosis and promotes a contractile VSMCs phenotype in the aorta of ApoE^−/−^ mice [97].

*MiR-133*. MiR-133, which is highly expressed in the vasculature, is downregulated after vascular injury and in proliferating VSMCs [98]. MiR-133 impairs the proliferation of VSMCs and inhibits the PDGF induced switch toward a synthetic VSMCs phenotype by repressing the transcription factor Sp-1. Accordingly, miR-133 is a key regulator of VSMCs phenotypic switch, suggesting its therapeutic potential for vascular diseases [98].

#### 3.2.2. MicroRNAs Mediating a Phenotype Switch toward Synthetic VSMCs

*MiR-21*. MiR-21 is upregulated after balloon injury and carotid ligation and promotes neointimal formation [99]. Inhibition of miR-21 decreases VSMCs proliferation and increases apoptosis; these effects are related to miR-21-induced activation of the Akt pathway via suppression of phosphatase and tensin homologue (PTEN) and upregulation of Bcl-2 expression [100]. In addition, miR-21 downregulates programmed cell death 4 (PDCD4), which in turn acts as a negative regulator of smooth muscle contractile genes [101, 102]. MiR-21 was significantly increased in human atherosclerotic plaques [103].

*MiR-221/222*. MiR-221 and miR-222 are augmented by vascular injury and increase neointimal formation through targeting the cell cycle inhibitors p27(Kip1) and p57(Kip2) [104]. PDGF pathway contributes to the neointimal proliferative responses through switching of VSMCs to synthetic phenotype. Phenotype switching of VSMCs by PDGF pathway is mediated by miR-221-induced repression of p27(Kip1) [105, 106]. MiR-221/222 participates in the regulation of angiogenesis in EPCs [107]. MiR-221/222 affects expression of c-kit, the receptor for stem cell factor, which plays a key role in EPCs migration and homing. Expression level of miR-221 in EPCs was upregulated in patients with CAD [108, 109]. Interestingly, miR-221 in EPCs was reduced in patients with CAD treated with atorvastatin [108, 109]. Moreover, miR-221 is induced under hyperglycemic conditions with impairing c-kit expression and migration of HUVECs [110]. Overexpression of miR-221 reduces the expression of eNOS [107].

*MiR-199a*. Recent study by Park et al. showed that expression of miR-199a/214 was downregulated in contractile SMCs, but significantly increased in synthetic SMCs. Inhibition of miR-199a showed dramatic upregulation of SM-*α*A in SMCs [111]. Interestingly, though these miRNAs are induced by SRF, phenotype switching by miR-199a/214 is mediated through activation of ELK1 and inhibition of SRF and myocardin [111].

## 4. Plaque Destabilization/Plaque Rupture and miRNAs

Vulnerable plaques are generally characterized by a lipid and vascular rich plaque with an overlying thin fibrous cap, containing numerous inflammatory macrophages and VSMCs. These findings are thought to be rupture prone characteristics leading to acute coronary syndrome in pathology [112].

*MiR-322*. Recently, a novel mouse model which can validate atherosclerotic plaque instability was developed [113]. Interestingly, this mouse has every stage of plaques from stable to unstable, or ruptured plaque, and the characteristics of plaques were quite similar to those of patients with CAD. Microarray analysis showed that miR-322 was upregulated in unstable plaques compared to stable plaques [113]. MiR-322 targets several genes including fibroblast growing factor 7 (FGF-7), CX3CL1, and FGF-1. Inhibition of miR-322 using anti-miR-322 showed a significant increase in FGF-7 expression. In addition, the expression of anti-inflammatory cytokine interleukin-10 (IL-10) was significantly upregulated, whereas the expression of proinflammatory cytokine IL-6 was significantly downregulated in response to miR-322 inhibition [113].

*MiR-125b and miR-204*. Vulnerable plaques often contain calcification in their necrotic cores. Several studies showed that miR-125b regulates the transdifferentiation of VSMCs into osteoblast-like cells by targeting the osteoblast transcription factor SP7 (Osterix) and Ets-1 [114, 115]. The expression of miR-125b was downregulated in calcified vessels in mouse and human, and osteogenic transdifferentiation of VSMCs was promoted by inhibition of miR-125b. MiR-204 was also found to be downregulated in aortic VSMCs during beta-glycerophosphate-induced calcification. Loss and gain of function experiments showed that miR-204 inhibited the VSMC calcification through downregulation of Runx2 [116]. Although there are no studies revealed that those miRNAs directly contributed to develop the vulnerable plaques, calcification is an important hallmark of artery atherosclerosis; therefore, these miRNAs regulating the VSMCs calcification may relate to plaque stability and have therapeutic potentials.

An observational study reported that expressions of miR-100, miR-127, miR-133a/b, and miR-145 were significantly increased in symptomatic plaques compared to asymptomatic plaques in human, indicating plaque instability [117]. Very recently, Fan et al. reported that expression of miR-21 in culture monocyte-derived macrophages is significantly increased in patients with noncalcified plaque compared to patients with calcified plaque or controls. They found that MMP-9 levels also correlate the expression of miR-21 and concluded that miR-21 participates in plaque instability by regulating MMP-9 via RECK in macrophages [118].

## 5. Platelet Function and miRNAs

Normal platelet functions such as activation, adhesion, and aggregation are essential for coagulation physiology and tissue repair. On the other hand, pathological platelet activation induces arterial thrombotic conditions such as myocardial infarction.

In patient with myocardial infarction, the exposure and release of plaque component following plaque rupture trigger the activation of platelets at the culprit lesion. Then, activated platelets release microparticles (MPs), carrying a broad variety of cytoplasmic components, including miRNAs, and are thought to be involved in subsequent process [119, 120]. Previous studies reported that human platelets contain an abundant and diverse array of microRNAs, and more than 400 miRNAs to date are detected [121, 122]. Little is known about the functions or its target genes of miRNAs in platelets. Recently, several studies investigated the biogenesis of specific miRNAs, and the role and importance of miRNAs in platelet function are gradually emerging.

*MiR-96*. MiR-96 was the first addressed miRNA involved in platelet reactivity in healthy individuals [123, 124]. In comparison to hyporeactivity platelets, hyperreactive platelets expressed higher levels of mRNA and protein of VAMP8 which is the target mRNA of miR-96. They demonstrated that overexpression of miR-96 in VAMP8-expressing cell lines caused dose dependent decrease in VAMP8 mRNA and protein upon expressing cell lines [123].

*MiR-223*. MiR-223 is one of the most abundant miRNAs in platelet and exists as a form of Ago2-miR-223 complex in platelet MPs. Human platelets activated by thrombin preferentially release Ago2-miR-223 complexes in MPs [125]. Interestingly, released Ago2-miR-223 complexes from MPs regulate the expression of endothelial mRNA: FBXW7 and EFNA1, indicating that activated platelets deliver mRNA regulatory Ago2-microRNA complexes to other cells and regulate expression of endogenous gene in recipient cell, such as ECs [125, 126]. More recently, miR-223 released from platelet is found to decrease the expression level of insulin-like growth factor 1 receptor in ECs and promoted HUVEC apoptosis [127]. In addition, miR-223 is predicted to regulate the expression of P2Y_12_, which is crucial for platelet aggregation, granule secretion, and thrombus growth and stability [122, 128, 129]. P2Y_12_ is also known as a receptor of thienopyridines including clopidogrel. In fact, miR-223 is found to be associated with platelet response to clopidogrel in patients with non-ST elevation ACS or healthy volunteers [130, 131]. After sufficient loading of clopidogrel and aspirin, platelet reactivity index (PRI) was measured and patients were divided into normal responders (PRI < 56.5%) and low responders (PRI > 56.5%). The expression of miR-223 is significantly downregulated in low responders [130].

These findings suggest that platelet miRNAs might reflect quantitatively platelet activation* in vivo* and, as such, might have a great potential as biomarkers of cardiovascular risk.

### 5.1. Other Platelet miRNAs

Gidlöf et al. reported that several platelet derived miRNAs were differentially expressed in patients with STEMI compared to those in controls [126]. Of these, they found that miR-320b released from activated platelets transfers to ECs and regulates the expression of ICAM-1 in ECs, indicating the paracrine role of miR-320b.

Microarray analysis revealed that several platelet-derived miRNAs such as miR-624^*^ and miR-340^*^ were significantly upregulated in patients with CAD compared to healthy controls [132]. It is well known that platelet also contributes to the development of chronic plaque formation as well as acute thrombotic event [133]. However, whether the expressions of these two miRNAs are cause or consequence of CAD is unrevealed. Further investigation of platelet miRNA may provide us with further understandings of the development of CAD.

### 5.2. Clinical Applications and Future Perspective

**Figure 1:**
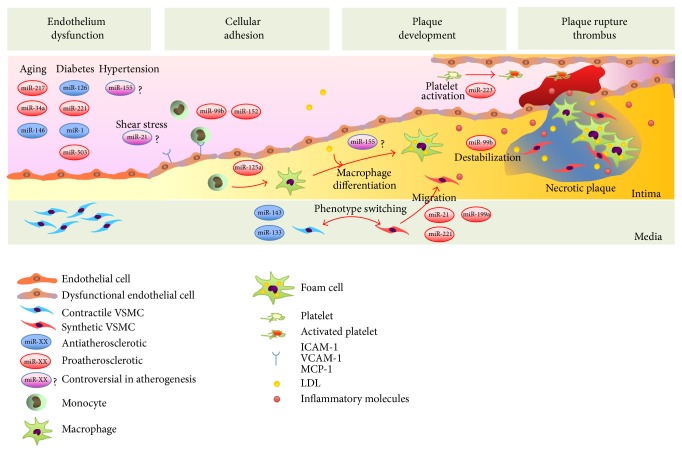
MicroRNAs in atherosclerotic plaque development, progression, rupture, and thrombus formation.

In this review, we summarized recent acknowledgement as possible as simple to understand the relation between miRNAs and atherosclerosis. Therefore, we mainly focused on vascular inflammation and plaque development (Figure 1). Atherosclerosis is a systemic disorder based on several disorders or factors; therefore there might be more miRNAs involving the development of CAD. For example, glucose metabolism and insulin resistance are deeply associated with pancreatic *β* cells, liver, and skeletal muscles, and recent studies have revealed the crucial role of miRNAs in glucose metabolism or development of T2D. Let-7 families, well known as tumor suppressor miRNAs, play an important role in glucose metabolism through regulating Lin-28 expression in muscles [134]. MiR-375 is a crucial miRNA for pancreatic island development, maintenance of pancreatic *α* cell and *β* cell functions, and insulin production [135–138]. Also, miR-143, miR-103, and miR-107 in liver are shown to be associated with glucose metabolism or insulin sensitivity, and abnormal expressions of these miRNAs are important mechanisms in the development of diabetes mellitus [139, 140].

Needless to say, lipid and cholesterol metabolism are also extensively associated with atherosclerosis, and recent studies have shown that miRNAs are critical contributors to regulate lipid or cholesterol homeostasis. MiR-122, mainly expressed in liver, is shown to affect hepatic lipid and cholesterol metabolism, and inhibition of miR-122 resulted in a reduction of cholesterol. MiR-33 regulates lipid and cholesterol homeostasis coordinating with sterol-regulatory element binding protein (SREBP) transcription factors, which are central players of lipid and cholesterol metabolism [141]. Recently, miR-33 deficiency is shown to reduce atherosclerotic plaque formation in mice [142].

In the past decade, a number of miRNAs are identified from tissues and continuously revealed the functions as described above. Now, it is obvious that dysregulation of miRNA expressions in each tissue directly links to the development of various diseases. Recently, miRNAs can be detected in plasma or serum as well as in tissues and called circulating miRNAs. Circulating miRNAs are resident in remarkably stable form within microparticles, Ago2 complexes, apoptosis body, and HDL to be protected from endogenous RNase activity [143]. In addition, many studies described the different profiles in circulating miRNAs between patients with CAD and healthy controls. For these advances, circulating miRNAs have a great potential as novel biomarkers.

MiR-208a and miR-1, which are muscle specific miRNAs, are the most promising miRNAs in early and accurate diagnosis of acute myocardial infarction (AMI) [144, 145]. These miRNAs are abundant in cardiac muscles and are seldom detectable in plasma in healthy controls. These miRNAs in circulation in patients with AMI are presumably originated from heart and reflect necrotic myocytes.

Moreover, circulating miR-133a and miR-208b in patients with AMI were associated with all-cause mortality at 6 months, also after adjustment for age and gender, indicating that miRNAs can also serve as biomarkers to predict the prognosis [146]. However, these miRNAs lost their association with outcome after adjustment for high sensitive TnT.

However, most studies were single center investigation based on relatively small samples. In addition, most studies were just analyzing the correlation of circulating level of miRNAs and disease. Whether the changes of circulating miRNA are cause or consequence is unanswered. It is necessary to reveal the detailed molecular mechanisms behind the change of circulating miRNAs, and to validate them in the global large scale studies, before using them as diagnostic biomarkers in clinical practice.

A number of studies have shown that miRNAs are essential regulators in biological activities. Loss or gain of functions experiments using chemically synthesized oligonucleotides shows potentials of miRNAs as novel therapeutic targets. There are two approaches: to decrease the activity (anti-miRs or inhibitors) or to increase the levels (pro-miRs or miR mimics). Although many problems need to be resolved to use in clinical setting, inhibitors of miR-208 and miR-15/195 family are candidate drugs to reduce heart failure or post-MI remodeling.

As an miRNA can bind to many mRNAs and regulate the protein expressions, unexpected effect is highly to occur. It is necessary to develop miR inhibitors or mimics which possess higher specificity, efficacy, and safety. It is necessary to develop optimized delivery strategies to target tissues.

## 6. Conclusions

Accumulating evidence has suggested that multiple miRNAs may serve as novel biomarkers and new therapeutic targets through their important roles in regulating cell proliferation, differentiation, and apoptosis. However, these potential roles of miRNA-based therapy should be further investigated. As a miRNA usually targets multiple genes and one gene may be targeted by multiple miRNAs, more studies are required to analyze the complex interactions between specific miRNAs and their target genes during CAD. It is very challenging to investigate the highly complex network of miRNAs. We believe that understanding the underlying mechanisms of miRNAs will provide novel opportunities for diagnosis and therapy of CAD.

## Figures and Tables

**Table 1:** Specific microRNAs regulating endothelial functions.

MicroRNA	Targets	Functions	Associated condition	References
Proatherosclerotic miRNA				
miR-221	c-kit	Reduces EPCs migraion and homing. Reduces eNOS expression	Diabetes or hyperglycemic condition	[107–109]
miR-503	cdc25, cyclin E	Inhibits cell cycle and differentiation	Diabetes or hyperglycemic condition	[29–31]
miR-217	SIRT-1	Induces senescent-like phenotype ECs	Aging	[58]
miR-34a				[59, 62–64]
miR-181b	importin-*α*3	Induces VCAM-1 and E-selection	Shear stress	[71]
Anti-atherosclerotic miRNA				
miR-126	SPRED1/VEGF	Reduces VCAM-1 expression	Diabetes or hyperglycemic condition	[13, 14]
miR-1	EDN-1	Reduces ET-1 expression	Diabetes or hyperglycemic condition	[27, 28]
miR-223	ICAM-1	Circulating HDL contains miR-223. Reduces ICAM-1 expression	Dyslipidemia and smoking	[33, 34, 49]
miR-146a	NOX4	Decreases SA*β*-gal positive cells	Aging	[66]
miR-10a	MAP3K7/*β*TRC	Reduces VCAM-1 and MCP-1 expression	Aging	[72]
Controversial miRNA				
miR-320	ET-1	Reduces ET-1, VEGF, and FN expression in HUVECs	Diabetes or hyperglycemic condition	[18]
		Inhibits ECs proliferation and migration in diabetc rats		[19]
miR-155	Ets-1/Ang II	Reduces Ang II-induced expression of VCAM-1 and Ang II type 1 recepter	Hypertension	[41–45]
		Reduces eNOS expression and NO production	/	[46, 47]
miR-21	PTEN	Increases eNOS expression and NO production	Shear stress	[67, 68]
	PPAR*α*	Induces VCAM-1 and MCP-1 expression		[69, 70]

EPCs: endothelial progenitor cells, eNOS: endothelial nitric oxide synthase, ECs: endothelial cells, VCAM-1: vascular cell adhesion molecule 1, SPRED1: sprouty-related protein 1, VEGF: vascular endothelial growth factor, ET-1: endothelin 1, EDN-1: ET-1 gene, HDL: high-density lipoprotein, ICAM-1: intracellular adhesion molecule 1, NOX4: NADPH oxidase-4, SA*β*-gal: senescence-associated *β*-galactosidase, MAP3K7: mitogen-activated kinase kinase kinase 7, *β*TRC: *β*-transducin repeat-containing gene, MCP-1: monocyte chemoattachment protein 1, FN: fibronectin, HUVECs human umbilical vein endothelial cells, Ang II: angiotensin II, PTEN: phosphatase and tensin homologue, PPAR*α*: peroxisome proliferators-activated receptor-α.
